# Effect of EEG-guided opioid-free anesthesia on perioperative blood glucose variation in patients undergoing video-assisted thoracic surgery: a secondary analysis of a randomized controlled study

**DOI:** 10.1186/s13741-026-00666-5

**Published:** 2026-03-03

**Authors:** Xiang Yan, Chen Liang, Meng Guo, Jia Jiang, Ying Ji, An-Shi Wu, Chang-Wei Wei

**Affiliations:** 1 Department of Anesthesiology, Beijing Chao-Yang Hospital, Capital Medical University, No. 8 Gongren Tiyuchang Nanlu, Chaoyang District, Beijing, 100020 China; 2 Department of Medical Statistics, Medieco Group Co., Ltd, Beijing, China; 3 Department of Thoracic Surgery, Beijing Institute of Respiratory Medicine and Beijing Chao-Yang Hospital, Capital Medical University, Beijing, China

**Keywords:** Opioid, Anesthesia, Glucose variability, Video-assisted thoracic surgery, Electroencephalogram

## Abstract

**Objectives:**

To evaluate the impact of electroencephalogram-guided opioid-free anesthesia (OFA) on perioperative blood glucose variability in patients undergoing video-assisted thoracic surgery.

**Methods:**

This article is a secondary analysis of a randomized controlled trial. Eligible patients were randomly allocated to either the OFA group or the opioid-based anesthesia (OBA) group. Patients in the OFA group did not receive any opioids during surgery, whereas those in the OBA group received sufentanil and remifentanil intraoperatively. The primary end point was the coefficient of variation for glucose (GLU_cv_), calculated based on predefined time points during the perioperative period. The secondary end points were the mean of glucose (GLU_mean_), the standard deviation of glucose (GLU_SD_), mean amplitude of glycemic excursions (MAGE), and largest amplitude of glycemic excursion (LAGE).

**Results:**

A total of 126 patients were included in the final analysis: the OFA group (*n* = 64) and the OBA group (*n* = 62). Blood pressure, heart rate, sedation index, and analgesia index during surgery were comparable between the two groups, and there was no significant difference in pain in the early postoperative period. The primary end point, GLU_CV_, was significantly higher in OFA group than in OBA group (22.7 ± 9.1 vs 14.1 ± 6.0, *P* < 0.001). In addition, the OFA group had higher GLU_mean_ (5.68 ± 0.87 vs 5.31 ± 0.87 mmol/L, *P* = 0.003), GLU_SD_ (1.3 ± 0.59 vs 0.76 ± 0.40 mmol/L, *P* < 0.001), MAGE (1.75 ± 0.86 vs 0.95 ± 0.54 mmol/L, *P* < 0.001), and LAGE (2.97 ± 1.35 vs 1.72 ± 0.96 mmol/L, *P* < 0.001).

**Conclusion:**

The OFA regimen provides sedation and analgesia comparable to OBA in video-assisted thoracic surgery, but is associated with greater perioperative blood glucose variation.

**Trial registration:**

Clinicaltrials.gov, Identifier: NCT05411159. First posted date: 9 Jun, 2022.

Date of enrolment of the first research participant: 10 Jun, 2022.

## Strengths and limitations of this study

- Previous research has shown that elevated blood glucose variability negatively impacts early postoperative recovery in patients. Specifically, patients undergoing opioid free anesthesia have been found to exhibit higher blood glucose levels during the procedure.
- To our knowledge, this is the first randomized controlled study to evaluate perioperative blood glucose variability in patients undergoing opioid free anesthesia.
- In this randomized controlled trial, we observed that patients who underwent surgery with opioid-free anesthesia exhibited higher blood glucose variability.
- Although the limited sample size in our study prevented a conclusive assessment of the link between elevated blood glucose variability and postoperative complications, the observed trend suggests that further investigation is warranted.

## Introduction

Opioid-free anesthesia (OFA) aims to reduce complications associated with opioid use and promote early recovery (Shanthanna et al. 2024). Currently, OFA programs are primarily applied in surgeries that involve low stimulation intensity, which sufficiently meets the analgesic requirements during the procedure (Silveira et al. 2024; Liu et al. 2024). However, it is unclear whether OFA can meet the analgesic needs of high-stimulation surgery, especially in thoracic surgery where perioperative pain stimulation is significant (Liu et al. 2023). Opioids exert paradoxical effects on glucose metabolism. They attenuate stress hyperglycemia by reducing sympathetic outflow and catecholamines, while also suppressing the hypothalamic–pituitary–adrenal axis to lower cortisol levels (Jain And Lai 2024). Conversely, studies in metabolically healthy individuals indicate that opioids can also promote insulin resistance (Byanyima et al. 2023). Previous studies indicated that patients receiving non-opioid anesthesia tend to experience an increase in blood glucose levels during surgery (An et al. 2021). Whether OFA leads to significant fluctuations in perioperative blood glucose levels is still uncertain.

The interaction of fasting, water deprivation, and surgical stress leads to significant fluctuations in perioperative blood glucose (Crowley et al. 2023). But in the hectic environment of operating rooms, blood glucose management often does not cause necessary attention. Perioperative hyperglycemia increases the risk of surgical site infection and poor wound healing (Li et al. 2021), while hypoglycemia increases the risk of cardiovascular events and even death. Managing blood glucose fluctuations is essential for effective perioperative care (Sebranek et al. 2013). Recent blood glucose management strategies have highlighted the importance of minimizing blood glucose variability as a key component of management (Ceriello et al. 2019; Monnier et al. 2023). Glycemic variability (GV) is an important measure for assessing fluctuations in blood glucose and has been identified as a significant contributing factor to the risk of death and vascular complications in patients with diabetes (Monnier et al. 2023; Egi et al. 2006; Krinsley 2008). In the context of perioperative management, it is vital to recognize the potential risks associated with blood glucose fluctuations.

Based on the above evidence, the aim of this study was to evaluate the impact of electroencephalogram-guided opioid-free anesthesia on perioperative blood glucose variability in patients undergoing video-assisted thoracic surgery.

## Methods

This study is a secondary analysis of a randomized controlled trial, and study protocol and the initially registered study outcomes have been made available to the public (Yan et al. 2023, 2025). The secondary analysis is based on exploratory purposes, therefore the calculation of GLU_CV_ was not described in the initial research plan. Both the initial research protocol and the secondary analysis protocol (2025-ke-466) have been approved by the ethics committee, and all patients signed informed consent forms.

### Participants

Patients aged 18–64 years, with a BMI of 18–35 kg/m2, ASA I-III, who underwent thoracoscopic lung surgery under general anesthesia from June 2022 to April 2023 were analyzed. Exclusion criteria: inability to communicate before surgery; receiving radiotherapy or chemotherapy within 14 days before surgery; using glucocorticoids within 14 days before surgery; expected postoperative mechanical ventilation time longer than 24 h; refusal to be included in the study. Exclusion criteria: surgery cancellation, postoperative loss to follow-up, withdrawal from the study, and missing blood glucose data.

### Randomization and masking

Subjects who signed the written informed consent form, after entering the operating room, were randomly assigned to the opioid-free anesthesia (OFA) group and the opioid-based anesthesia (OBA) group in a 1:1 ratio using pre-prepared sealed envelopes. Only the anesthesiologists managing the intraoperative anesthesia were aware of the group allocation, while the patients and researchers conducting the follow-up for the endpoint outcomes were blinded.

### Anesthesia, perioperative care, and intervention

All subjects were required to abstain from water for 4 h and fast for 8 h before surgery. Electrocardiogram, arterial blood pressure, and finger oxygen saturation were monitored during surgery. The targeted blood pressure during surgery was maintained within ± 25% of baseline, with vasoactive agents administered as necessary. Spontaneous Electroencephalogram (EEG) wavelet was monitored using the HXD-I multifunction combination monitor (Heilongjiang Huaxiang Technology Co., Ltd., Heilongjiang, China). The EEG-derived wavelet index (WLi), which ranges from 0 to 100 (a lower value indicates greater degree of sedation), was used to assess the depth of sedation, with a target WLi value set between 40 and 60 during the operation. The EEG-derived pain threshold index (PTi), also ranging from 0 to 100 (a lower value indicates greater pain tolerance), was targeted to less than 65 during surgery. All patients received a double-lumen endotracheal for one-lung ventilation.

All patients were given midazolam 0.025 mg/kg for sedation and dexamethasone 5 mg to prevent postoperative nausea and vomiting after entering the operating room. Before incision, both groups received an ultrasound-guided thoracic paravertebral nerve block with a single injection of 0.5% ropivacaine 20 ml at T_4–5_ level on the dependent side. Additionally, flurbiprofen 50 mg was administered intravenously before the incision and at the time of sutures. According to the allocation, patients received different anesthesia regimens.

In the OFA group, dexmedetomidine was infused at a rate of 1 µg/kg for 10–15 min prior to induction. Anesthesia was then induced using propofol (1.5–2.5 mg/kg), rocuronium (0.6 mg/kg), and lidocaine (1.5 mg/kg). Maintenance included dexmedetomidine (0.5 µg/kg/h), desflurane (0.5 MAC), and propofol (2–4 mg/kg/h) until the end of the suturing process.

In the OBA group, induction involved propofol (1.5–2.5 mg/kg), rocuronium (0.6 mg/kg), and sufentanil (0.3 µg/kg). Maintenance was achieved with remifentanil (0.1–0.3 µg/kg/min), desflurane (0.5 MAC), and propofol (2–3 mg/kg/h).

### Measurements

The characteristics of surgery and anesthesia was recorded, including the surgery type, surgery duration, intraoperative medication, and fluid balance. To evaluate the analgesic effect of OFA versus OBA. The 8 key time points for analysis were established: baseline, intubation, skin incision, one-lung ventilation start, one-lung ventilation end, skin suture, extubation, and operating room discharge. Additionally, numeric rating scale (NRS) scores of pain at rest were recorded at 15 min, 1 h, and 24 h after surgery. Patients were followed up after surgery until discharge, and complications within 7 days were recorded and graded by Clavien-Dindo Classification (Dindo et al. 2004).

The perioperative blood glucoses of the patients were measured at 4 key time points: baseline (between 6:00 and 8:00 a.m. at surgery day), intubation (within 5 min after successful intubation), lesion resection (within 5 min after resection), and day 1 (between 6:00 and 8:00 a.m. at the postoperative day 1). Blood samples were obtained from the patient's elbow vein. Blood glucose was measured using an ADVIA® 2400 biochemical analyzer (Siemens Healthcare Diagnostics, Eschborn, Germany).

### Study endpoints

The primary endpoint for perioperative glucose fluctuations was the coefficient of variation for glucose (GLU_CV_ = [standard deviation of glucose/mean glucose] × 100%) (Monnier et al. 2023), which quantifies glucose variability through repeated perioperative blood glucose measurements. Secondary endpoints of glycemic variability included the mean of glucose (GLU_mean_), the standard deviation of glucose (GLU_SD_), mean amplitude of glycemic excursions (MAGE), and largest amplitude of glycemic excursion (LAGE) (Monnier et al. 2018; Wang et al. 2022). Based on the geographical characteristics of the subject, GLU_CV_ > 33% was considered indicative of high GV (Mo et al. 2021).

### Statistics

The Shapiro–Wilk test was employed to assess the normality of continuous variables. Continuous variables with a normal distribution were presented as mean ± standard deviation (mean ± standard deviation), and an independent samples t-test was conducted for comparisons between groups. For continuous variables without a normal distribution, results were expressed as median and interquartile range (Median [quartile]), with the Mann–Whitney U test used for group comparisons. Categorical data were reported as the number of cases (%), using either the Chi-Square test or Fisher's exact test for group comparisons. Balance between groups at baseline was evaluated using the absolute standardized difference.

For the primary and second endpoints of glycemic variability were analyzed using a t-test for group comparisons, with the mean difference (MD) and 95% confidence intervals (CI) reported. Other secondary and exploratory outcomes were analyzed by *χ*^*2*^ test, Student t-tests, or *Mann–Whitney U* test as appropriate. For repeated measurement data, such as blood glucose, blood pressure, heart rate, WLi, and PTi, repeated measures analysis of variance was used for overall comparisons. Mauchly’s test was used to assess sphericity, and we examined the distribution of residuals to ensure normality. The Bonferroni method was applied to adjust *p*-values for multiple comparisons between groups at each time point. Statistical significance was defined as *P* < 0.05. No imputation was performed for missing data. Data analysis was performed using SPSS 26.0 software.

### Sample size estimation

Based on data from previous studies, the mean and standard deviation of the percentage coefficient of variation in blood glucose for the OBA group were assumed to be 15% and 6%, respectively. Additionally, the coefficient of variation for blood glucose in the OFA group was expected to be at least 5 points higher compared to the control group. With a significance level (α) of 0.05 and a power (1-β) of 0.9, a minimum of 32 patients were required in each group.

## Results

**Fig. 1:**
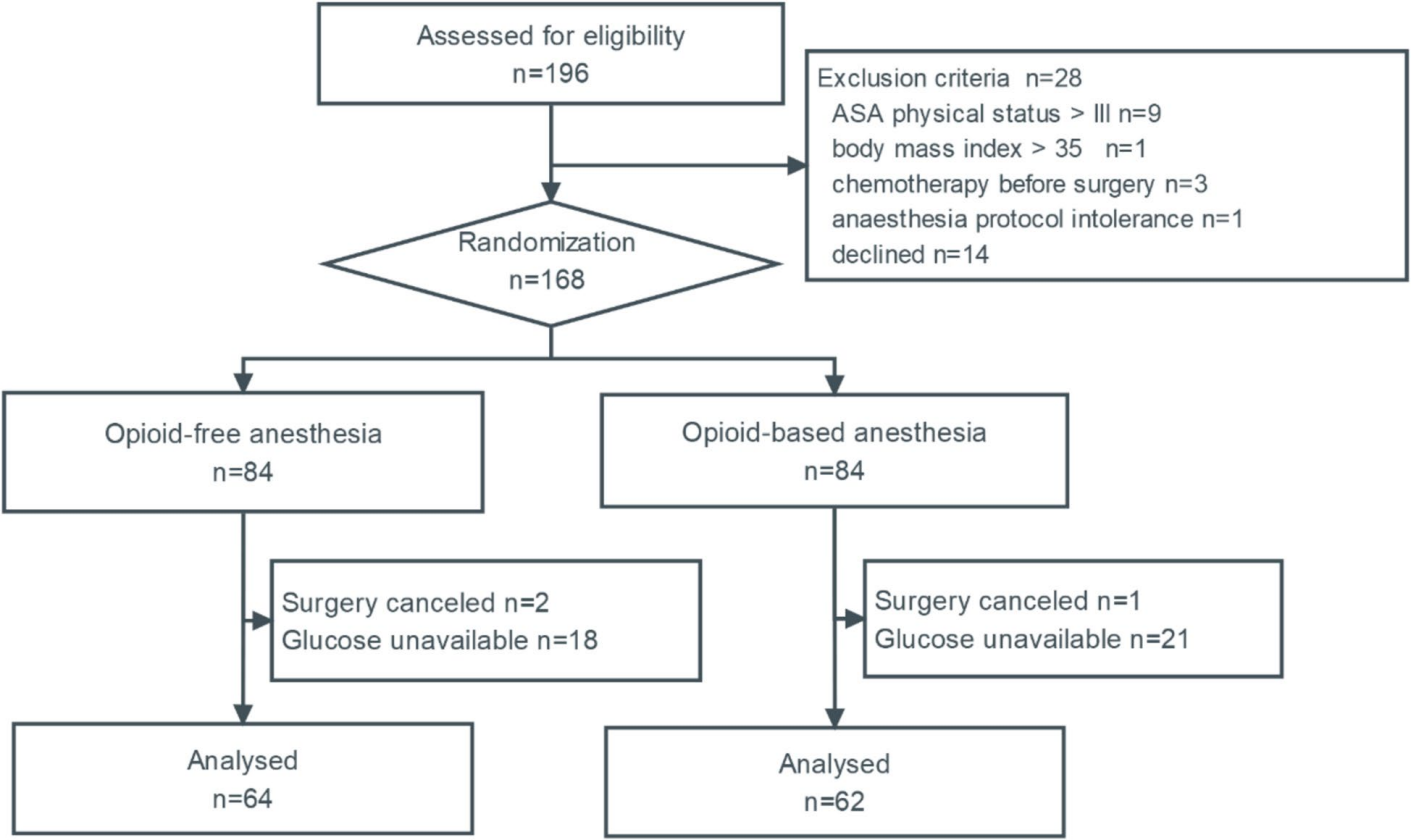
Flowchart

A total of 126 subjects who completed the measurements of blood glucose within the four specified time frames were included in the final analysis. (Fig. 1) No patient received intraoperative glucose-containing solutions or glucocorticoids. There were no significant differences in demographic characteristics, comorbidity, proportion of diabetes, surgery duration, or anesthesia duration between the two groups (Table 1).

**Table 1:** Baseline, surgical, and anesthetic characteristics

	**OFA group** ***n*** ** = 64**	**OBA group** ***n*** ** = 62**	**absolute standard difference**	***P*** ** value**
Age, years	54 ± 9	55 ± 9	0.111	0.756
Female	36 (56.3%)	42 (67.7%)	0.237	0.184
BMI	24.5 ± 3.4	24 ± 3.5	0.145	0.225
ASA I/II/III	22/39/3	27/32/3	0.187	0.190
Smoking	14 (21.9%)	10 (16.1%)	0.148	0.412
Drinking	9 (14.1%)	4 (6.5%)	0.252	0.160
Comorbidity				
Hypertension	21 (32.8%)	15 (24.2%)	0.191	0.284
Hyperlipidemia	16 (25.0%)	15 (24.2%)	0.019	0.916
Coronary heart disease	2 (3.1%)	3 (4.8%)	0.087	0.622
COPD	3 (4.7%)	1 (1.6%)	0.178	0.619
Diabetes	10 (15.6%)	8 (12.9%)	0.055	0.663
Biguanides	9 (14.1%)	7 (11.3%)	0.083	0.640
Sulfonylureas	4 (7.8%)	2 (3.2%)	0.202	0.262
Glinides	2 (3.1%)	1 (1.6%)	0.099	0.512
Insulin	1 (1.6%)	0	0.178	0.508
Charlson Comorbidity index	1 [1, 2]	2 [1, 2]	0.162	0.348
Preoperative hemoglobin	139 ± 13	135 ± 15	0.285	0.125
Preoperative blood glucose	5.08 ± 1.09	5.17 ± 1.16	0.080	0.735
FEV1/FVC	76.6 ± 6.1	76.6 ± 5.9	0.001	0.996
Lung cancer	62 (96.9%)	59 (95.2%)	0.087	0.755
Lesion side left/right	27/37	25/37	0.038	0.832
Surgery type			0.037	0.304
Wedge resection	29 (45.3%)	27 (43.5%)		
Segmentectomy	11 (17.2%)	18 (29.0%)		
Lobectomy	24 (37.5%)	17 (27.4%)		
Surgery duration	101 ± 46	106 ± 53	0.101	0.790
Anesthesia duration	130 ± 47	134 ± 55	0.078	0.911
Anesthetics				
Sufentanil, ug	-	20 ± 5	-	-
Remifentanil, ug	-	1264 ± 575	-	-
Dexmedetomidine, ug	70 ± 20	-	-	-
Propofol, mg	499 ± 153	464 ± 167	0.155	0.088
Desflurane concentration, %	0.5 ± 0	0.5 ± 0	0.001	0.310
Rocuronium, mg	61 ± 15	58 ± 14	0.207	0.059
Vasopressor used	9 (14.1%)	14 (22.6)	0.226	0.148
Fluid balance				
Crystalloid solution, ml	1239 ± 326	1239 ± 329	0.012	0.527
4% hydroxyethyl starch, ml	109 ± 208	121 ± 234	0.054	0.887
Blood loss, ml	23 ± 20	22 ± 18	0.052	0.915
Urine volume, ml	341 ± 151	365 ± 180	0.147	0.922

Data are mean ± standard deviation, median [inter-quartile range], or n (%)

*OFA* opioid-free anesthesia, *OBA* opioid-based anesthesia. *ASA* American Society of Anesthesiologists, *BMI* body mass index, *COPD* Chronic Obstructive Pulmonary Disease, *FEV1/FVC* Forced Expiratory Volume in 1 s/Forced Vital Capacity

### Primary outcome

**Fig. 2:**
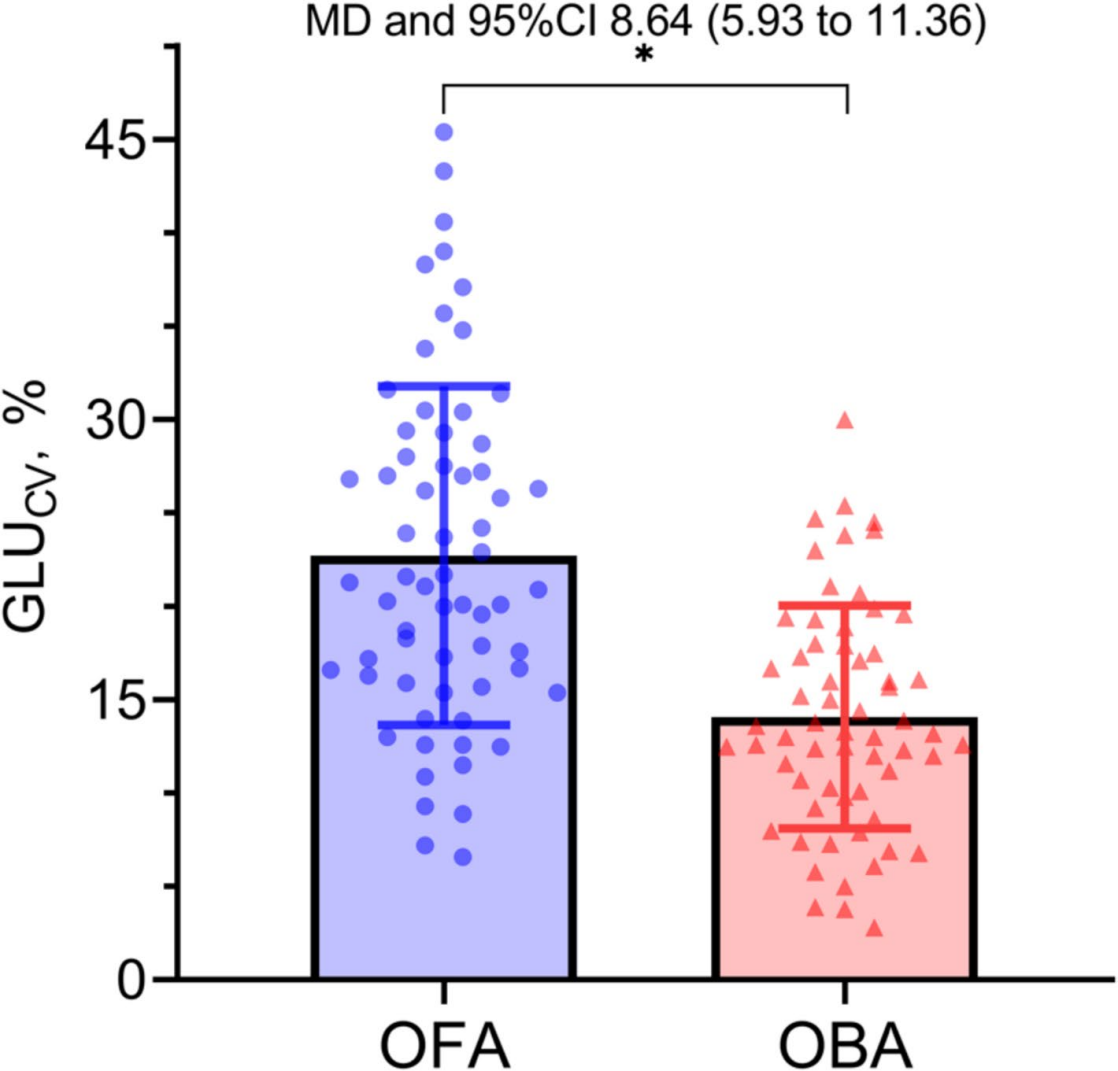
Column scatter plot of coefficient of variation for glucose (GLU_CV_). The bars and error bars represent the mean and standard deviation, respectively. Solid circles depict data distribution in the opioid-free anesthesia (OFA) group, while solid triangles indicate the opioid-based anesthesia (OBA) group. MD, mean difference. CI: confidence interval. Asterisks (*) indicate statistical significance, with *P* < 0.05

The glycemic variability, GLU_CV_, was significantly higher in OFA group than that in OBA group (22.7 ± 9.1 vs 14.1 ± 6.0, *P* < 0.001). (Table 2) (Fig. 2).

**Table 2:** Primary and secondary endpoints

	**OFA ** ***n*** ** = 64**	**OBA ** ***n*** ** = 62**	**Effect size (95% CI)**	***P*** ** value**
Primary endpoint				
Coefficient of variation for glucose, %	22.7 ± 9.1	14.1 ± 6.0	MD 8.64 (5.93 to 11.36)	< 0.001
Secondary endpoint				
mean of glucose	5.68 ± 0.87	5.31 ± 0.87	MD 0.37 (0.06 to 0.68)	0.003
standard deviation of glucose	1.30 ± 0.59	0.76 ± 0.40	MD 0.54 (0.36 to 0.72)	< 0.001
mean amplitude of glycemic excursions	1.75 ± 0.86	0.95 ± 0.54	MD 0.80 (0.55 to1.05)	< 0.001
largest amplitude of glycemic excursion	2.97 ± 1.35	1.72 ± 0.96	MD 1.25 (0.83 to 1.67)	< 0.001
Coefficient of variation for glucose > 33%	9 (14.1%)	0 (0%)	-	0.003
NRS score for pain at rest				
15min	1 [0,1]	1 [1,1]	MD −0.05 (−0.38 to 0.28)	0.485
1h	1 [1,2]	1 [1,2]	MD 0.08 (−0.28 to 0.44)	0.727
24h	3 [2,4]	3 [2,3]	MD −0.11 (−0.57 to 0.36)	0.582
Postoperative complication by Clavien-Dindo grade			-	0.060
I	1 (1.6%)	5 (8.1%)		
II	2 (3.1%)	0		
IIIa	0	0		
IIIb	1 (1.6%)	0		
≥ IV	0	0		
Length of Hospital Stay, day	3 [2,5]	3 [2,4]	MD 0.15 (−0.46 to 0.75)	0.645

Data are mean ± standard deviation, median [inter-quartile range], or n (%)

*OFA* opioid-free anesthesia, *OBA* opioid-based anesthesia, *MD* mean difference. *CI* confidence interval, *NRS* Numerical Rating Scale

### Secondary outcomes

Compared with the OBA group, the OFA group had higher GLU_mean_ (5.68 ± 0.87 vs 5.31 ± 0.87 mmol/L, *P* = 0.003), GLU_SD_ (1.3 ± 0.59 vs 0.76 ± 0.40 mmol/L, *P* < 0.001), MAGE (1.75 ± 0.86 vs 0.95 ± 0.54 mmol/L, *P* < 0.001), and LAGE (2.97 ± 1.35 vs 1.72 ± 0.96 mmol/L, *P* < 0.001). For the perioperative glucose fluctuations, the incidence of hyperglycemia variability (GV > 33%) was significantly higher in the OFA group than in the OBA group (14.1% vs. 0%, *P* = 0.003) (Table 2). Repeated measures analysis of variance was performed on the glucose at the 4 time points, and the overall difference between the OFA group and the OBA group was statistically significant (F = 5.621, *P* = 0.019). The blood glucose level after lesion resection in OFA group was higher than that in OBA group (7.36 ± 1.47 vs. 5.94 ± 1.03, Bonferroni adjusted *P* = 0.001), but the difference was not statistically significant at other time points.

**Fig. 3:**
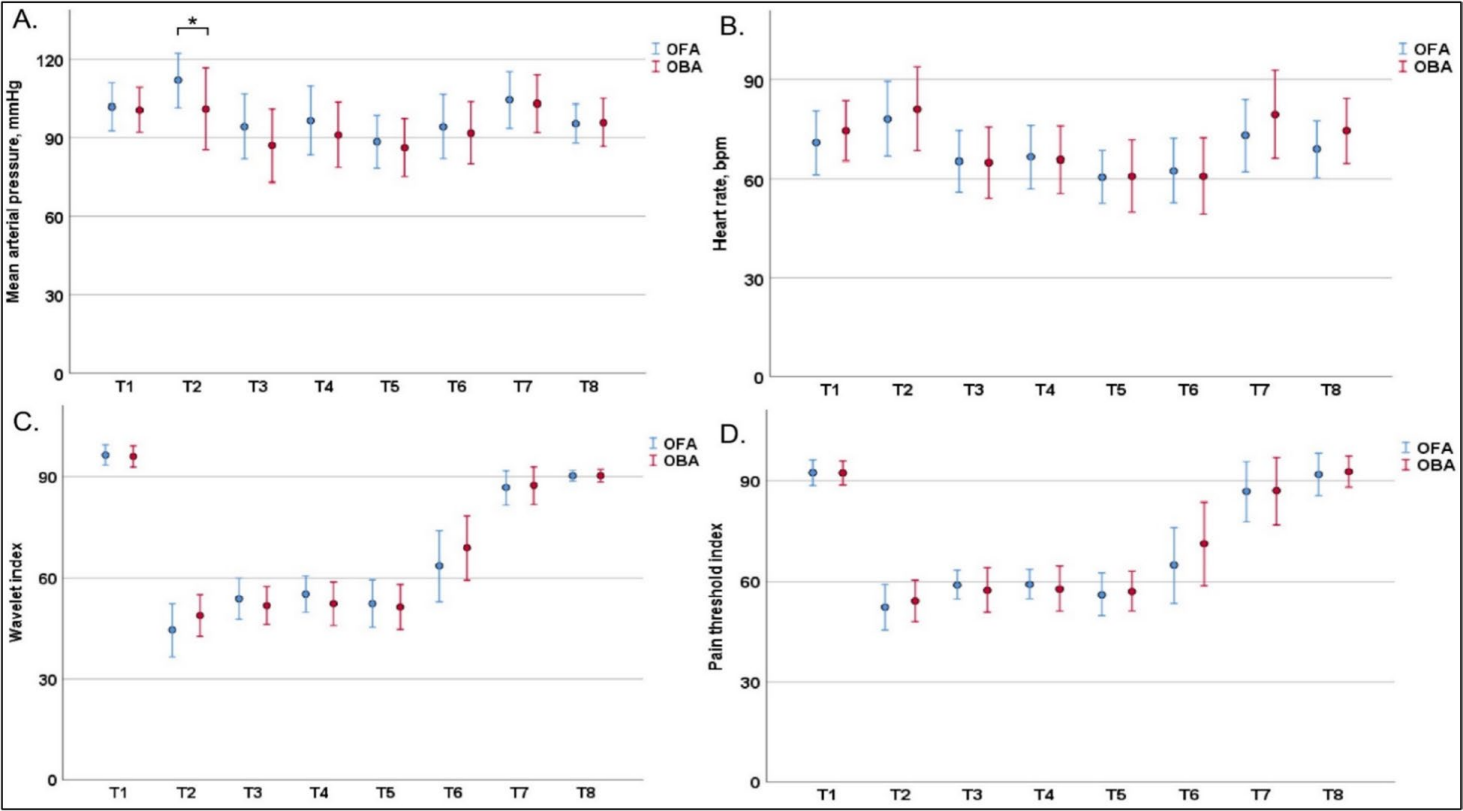
Clustered error bar mean graphs of blood pressure (**A**), heart rate (**B**), WLi (**C**), and PTi (**D**) at 8 time points: T1 (baseline), T2 (intubation), T3 (skin incision), T4 (one-lung ventilation start), T5 (one-lung ventilation end), T6 (skin suture), T7 (extubation), and T8 (operating room discharge). The dots and error bars represent the mean values and standard deviation, respectively. OFA, opioid-free anesthesia group; OBA, opioid-based anesthesia group. Asterisks (*) indicate statistical significance, with Bonferroni adjusted *P* < 0.05

At the 8 time points in the operating room, the WLi (F = 0.844, *P* = 0.360), PTi (F = 1.945, *P* = 0.166), and heart rate (F = 1.085, *P* = 0.300) showed no significant difference between the two groups. There was a significant difference in intraoperative blood pressure between the two groups (F = 12.519, *P* = 0.001), which was due to the higher mean arterial pressure during bronchial intubation in the OFA group than in the OBA group (112 ± 10 vs. 101 ± 16 mmHg, Bonferroni adjusted *P* = 0.002) (Fig. 3). Postoperative NRS scores for pain at rest were comparable at 15 min, 1 h, and 24 h after surgery (Table 2).

## Discussion

In this study, we found that EEG-guided OFA provided similar levels of sedation depth, analgesia index, heart rate, and blood pressure during most time points of surgery, as well as comparable postoperative pain scores when compared to OBA for patients undergoing video-assisted thoracic surgery. However, we observed a significant increase in glucose variability in the OFA group, primarily due to changes in blood glucose levels during the operation. Therefore, it is recommended that attention be given to fluctuations in blood glucose during thoracic surgery when using the OFA regimen, which may pose a potential risk.

The feasibility of using an OFA has been demonstrated in minimally invasive surgeries with moderate stimulation intensity (Silveira et al. 2024; Liu et al. 2024). However, in thoracic surgery, where stimulation is stronger, the optimal OFA protocol is still being explored (Shanthanna et al. 2024). In this study, we observed that in the OFA group, the mean arterial pressures for most patients were within the target range but higher than those in OBA group during bronchial intubation. In the future practice, more measures should be taken to reduce the stimulation during bronchial intubation in OFA protocol. There were no significant differences in blood pressure, heart rate, depth of sedation, or pain index between the two groups during the thoracic procedure. Additionally, early pain NRS scores showed no significant differences. This is consistent with the results of a recent study (An et al. 2021). Overall, our results indicate that the OFA regime provides an analgesic effect comparable to OBA protocols during operation.

An’s study observed that patients in the OFA group experienced higher blood glucose levels during surgery (An et al. 2021). Compared with transient hyperglycemia, elevated GV shows a stronger correlation with postoperative complications (Chen et al. 2024). In this study, patients in the OFA group exhibited higher variability based on predefined time points during the perioperative period. Drastic fluctuations in blood glucose levels during the perioperative period can significantly increase the risk of vascular-related complications (Brownlee And Hirsch 2006). High perioperative GV has been identified as an independent risk factor for poor patient prognosis. Several cohort studies with large sample sizes found that high GV after surgery is strongly associated with prolonged hospital stays and an increased risk of mortality (Egi et al. 2006; Krinsley 2008). Additionally, high GV is linked to a greater incidence of postoperative complications. One study indicated that high GV elevates the risk of postoperative acute kidney injury (odds ratio [OR] = 1.58) (Lee et al. 2021). Moreover, the overall risk of short-term adverse outcomes—such as death, secondary surgery, deep sternal infection, stroke, pneumonia, renal failure, pericardial tamponade, and myocardial infarction—was also increased (OR = 1.91) (Rangasamy et al. 2020). After discharge, patients with high GV had higher readmission rates (OR = 2.19), a greater likelihood of surgical site infections (OR = 3.22), and an increased risk of reoperation (OR = 2.65) (Canseco et al. 2022). The above evidence suggests that high GV increases the risk of poor surgical prognosis, but the attention during the perioperative period still needs to be improved (Crowley et al. 2023). Glucose fluctuations can cause damage through several potential mechanisms. The hyperglycemia may lead to carotid intima thickening and increased ventricular mass through oxidative stress, circulating inflammatory cytokine levels, and subsequent endothelial dysfunction (Frontoni et al. 2013). These factors contribute to a higher risk of death, particularly in patients with diabetes. Furthermore, fluctuations in hyperglycemia may disrupt "metabolic memory" in vascular cells (Schisano et al. 2011), reducing their antioxidant capacity (Frontoni et al. 2013). Even in the healthy adults, acute hyperglycemia has been shown to downregulate various genes responsible for detoxifying free radicals (Meugnier et al. 2007). Although higher blood glucose fluctuations were observed in the OFA group patients in this study, there was no significant increase in the incidence of postoperative complications. This may require larger sample size studies to confirm whether high GV during such surgeries has significant clinical significance.

To the best of our knowledge, this is the first randomized controlled study to indicate that opioid-free anesthesia increases perioperative GV. There are several limitations to this study. First, since it was a randomized controlled trial, we could not blind the anesthesiologists administering the different anesthesia regimens. Nevertheless, the primary outcome of the study—blood glucose—remains an objective evaluation metric, thus reducing the potential bias. In assessing postoperative pain, both participants and follow-up personnel were blinded to minimize possible bias. Second, our evaluation of the intraoperative analgesic effect may have some limitations. We utilized blood pressure and heart rate indicators, along with EEG-derived sedation and analgesia index, for intraoperative management. Additionally, the assessment of the postoperative pain intensity by using NRS scores is subjective, but this method remains an indirect reflection of intraoperative pain stimulation. Future studies could benefit from more objective indicators, such as stress-related hormones or indicators of inflammatory factors. Finally, we assessed the impact of OFA on blood GV by sampling at key stimulus time points during the perioperative period. However, this approach may not adequately represent the entire dynamic evolution of blood glucose in patients throughout the perioperative phase. Future research employing continuous glucose monitoring during surgery would be beneficial for providing a more detailed understanding of the dynamic changes in blood glucose levels.

## Conclusions

In conclusion, opioid-free anesthesia regimens provide patients with sedative and analgesic effects comparable to those of opioid-based anesthesia during thoracoscopic surgical procedures. However, intraoperative and postoperative short-term blood glucose measurements indicated that patients in the opioid-free anesthesia group may exhibited higher levels of blood glucose variability. Given the potential risk of increased variability in hyperglycemia, future studies with more extensive time points and longer durations are needed to better understand the impact of anesthesia regimens on glucose variability.

## Data Availability

The datasets used and analyzed during the current study are available from the corresponding author upon reasonable request.

## Abbreviations

ASA: American Society of Anesthesiologist
BMI: Body mass index
EEG: Electroencephalogram
GLU_CV_: Coefficient of variation for glucose
GLU_mean_: Mean of glucose
GLU_SD_: Standard deviation of glucose
GV: Glycemic variability
IQR: Inter-quartile range
LAGE: Largest amplitude of glycemic excursion
MAGE: Mean amplitude of glycemic excursions
MD: Mean differences
NRS: Numerical Rating Scale
OBA: Opioid-based anesthesia
OFA: Opioid-free anesthesia
OR: Odds ratio
SD: Standard deviation
PTi: Pain threshold index
WLi: Wavelet index
